# Cerebrospinal Fluid Neurofilament Light Predicts Risk of Dementia Onset in Cognitively Healthy Individuals and Rate of Cognitive Decline in Mild Cognitive Impairment: A Prospective Longitudinal Study

**DOI:** 10.3390/biomedicines10051045

**Published:** 2022-04-30

**Authors:** Kunal Dhiman, Victor L. Villemagne, Christopher Fowler, Pierrick Bourgeat, Qiao-Xin Li, Steven Collins, Ashley I. Bush, Christopher C. Rowe, Colin L. Masters, David Ames, Kaj Blennow, Henrik Zetterberg, Ralph N. Martins, Veer Gupta

**Affiliations:** 1 IMPACT—The Institute for Mental and Physical Health and Clinical Translation, School of Medicine, Deakin University, 75 Pigdons Rd, Geelong, VIC 3216, Australia; kunal.dhiman@deakin.edu.au; 2 Western Health Partnership, School of Nursing and Midwifery, Faculty of Health, Deakin University, Melbourne, VIC 2600, Australia; 3 School of Medical and Health Sciences, Edith Cowan University, 270 Joondalup Drive, Joondalup, WA 6027, Australia; r.martins@ecu.edu.au; 4 Department of Psychiatry, University of Pittsburgh, Pittsburgh, PA 15213, USA; victorlv@unimelb.edu.au; 5 Department of Molecular Imaging & Therapy and Centre for PET, Austin Health, Melbourne, VIC 3084, Australia; crowe@unimelb.edu.au; 6 Department of Medicine, The University of Melbourne, Melbourne, VIC 3010, Australia; steven.collins@florey.edu.au; 7 The Florey Institute of Neuroscience and Mental Health, The University of Melbourne, Melbourne, VIC 3010, Australia; christopher.fowler@florey.edu.au (C.F.); qiao-xin.li@florey.edu.au (Q.-X.L.); ashley.bush@florey.edu.au (A.I.B.); c.masters@florey.edu.au (C.L.M.); 8 Australian e-Health Research Centre, CSIRO Health and Biosecurity, Brisbane, QLD 4029, Australia; pierrick.bourgeat@csiro.au; 9 Co-Operative Research Centre for Mental Health, Melbourne, VIC 3053, Australia; 10 National Ageing Research Institute, Melbourne, VIC 3052, Australia; dames@unimelb.edu.au; 11 Academic Unit for Psychiatry of Old Age, St. George’s Hospital, The University of Melbourne, Melbourne, VIC 3010, Australia; 12 Department of Psychiatry and Neurochemistry, Institute of Neuroscience and Physiology, The Sahlgrenska Academy at the University of Gothenburg, 43180 Mölndal, Sweden; kaj.blennow@neuro.gu.se (K.B.); henrik.zetterberg@clinchem.gu.se (H.Z.); 13 Clinical Neurochemistry Laboratory, Sahlgrenska University Hospital, 43180 Mölndal, Sweden; 14 Department of Neurodegenerative Disease, UCL Queen Square Institute of Neurology, Queen Square, London WC1E 6BT, UK; 15 UK Dementia Research Institute at UCL, London WC1E 6BT, UK; 16 Hong Kong Center for Neurodegenerative Diseases, Clear Water Bay, Hong Kong, China; 17 Australian Alzheimer’s Research Foundation, Ralph and Patricia Sarich Neuroscience Research Institute, 8 Verdun Street, Perth, WA 6009, Australia; 18 Department of Biomedical Sciences, Macquarie University, Sydney, NSW 2109, Australia; 19 School of Psychiatry and Clinical Neurosciences, University of Western Australia, Perth, WA 6009, Australia; 20 KaRa Institute of Neurological Diseases, Sydney, NSW 2113, Australia

**Keywords:** neurofilament light, cerebrospinal fluid, preclinical, early diagnosis, prognosis

## Abstract

Background: Biomarkers that are indicative of early biochemical aberrations are needed to predict the risk of dementia onset and progression in Alzheimer’s disease (AD). We assessed the utility of cerebrospinal fluid (CSF) neurofilament light (NfL) chain for screening preclinical AD, predicting dementia onset among cognitively healthy (CH) individuals, and the rate of cognitive decline amongst individuals with mild cognitive impairment (MCI). Methods: Neurofilament light levels were measured in CSF samples of participants (CH, *n* = 154 and MCI, *n* = 32) from the Australian Imaging, Biomarkers and Lifestyle study of ageing (AIBL). Cases of preclinical AD were identified using biomarker-guided classification (CH, amyloid-β [Aβ]+, phosphorylated-tau [P-tau]+ and total-tau [T-tau]±; A+T+/N±). The prediction of dementia onset (questionable dementia) among CH participants was assessed as the risk of conversion from Clinical Dementia Rating [CDR = 0] to CDR ≥ 0.5 over 6 years. Mixed linear models were used to assess the utility of baseline CSF NfL levels for predicting the rate of cognitive decline among participants with MCI over 4.5 years. Results: Neurofilament light levels were significantly higher in preclinical AD participants (CH, A+T+/N±) as compared to A-T-N- (*p* < 0.001). Baseline levels of CSF NfL were higher in CH participants who converted to CDR ≥ 0.5 over 6 years (*p* = 0.045) and the risk of conversion to CDR ≥ 0.5 was predicted (hazard ratio [HR] 1.60, CI 1.03–2.48, *p* = 0.038). CH participants with CSF NfL > cut-off were at a higher risk of developing dementia (HR 4.77, CI 1.31–17.29, *p* = 0.018). Participants with MCI and with higher baseline levels of CSF NfL (>median) had a higher rate of decline in cognition over 4.5 years. Conclusion: An assessment of CSF NfL levels can help to predict dementia onset among CH vulnerable individuals and cognitive decline among those with MCI.

## 1. Introduction

Alzheimer’s Disease (AD) is a disease of multiple etiologies [1]. The associated neuropathological aberrations initiate at the preclinical stage—a “silent” stage of the AD continuum devoid of symptoms of cognitive decline, but with evident biochemical changes in the brain [2]. Preclinical AD is classified into three stages based on amyloid status, neuronal injury and the presence of subtle cognitive change [3]. Stage one is categorized as the stage of “asymptomatic cerebral amyloidosis”, with evidence of brain amyloidosis, and stage two presents evidence of both amyloidosis and neurodegeneration [3]. This staging aligns with the recently proposed ATN criteria in which individuals with suspected AD can be classified based on whether they present biomarker evidence of amyloid pathology (A), tau pathology (T) and neurodegeneration (N) [4]. Pathophysiological biomarkers indicative of early biochemical aberrations in the brain are needed to identify individuals who are in the preclinical stage or those likely at risk of developing dementia.

Neurodegeneration initiates early in the AD continuum, believed to be triggered by several pathological changes, such as amyloidopathy, tauopathy, neuroinflammation and oxidative stress [5]. Therefore, pathophysiological biomarkers of neurodegeneration should be explored and evaluated for their ability to screen preclinical AD, predict dementia onset in cognitively healthy (CH) aged individuals and disease progression (decline in cognition) in those with MCI. Cerebrospinal fluid (CSF) neurofilament light (NfL) chain is a biomarker of neurodegeneration whose levels are elevated in AD and associated with central AD neuropathology [6,7,8,9,10]. Neurofilament proteins, including NfL, act as axonal supports, are vital for axonal growth and impulse conduction [11] and are shed during axonal degeneration. Elevated levels of CSF NfL associate with cognitive impairment and brain atrophy over time [6]. Several studies have indicated the potential of plasma NfL for diagnosing AD [12,13,14,15], thus creating a pathway for the development of a non-invasive diagnostic test for AD. Although NfL non-specifically increases in several neurological diseases, it has the potential to differentiate AD from other dementias, particularly frontotemporal dementia (FTD) [16], which has also been validated in meta-analyses conducted by Bridel et al. [17] and Forgrave et al. [18].

In addition to its diagnostic utility for AD, NfL can be used as a screening biomarker of early neurodegeneration to predict the risk of dementia onset in the vulnerable population, as well as a prognostic biomarker of downstream neurodegenerative changes to identify cognitively impaired individuals who are likely to progress further along the trajectory of cognitive impairment. Studies indicate that elevated levels of CSF NfL predict the risk of conversion to mild cognitive impairment (MCI), among CH participants [19], and correlate with cognitive decline, white matter changes and brain atrophy in participants with MCI [6]. Previously, we confirmed the diagnostic potential of CSF NfL in AD, and the association of CSF levels with central AD neuropathology and cognition [8]. To further evaluate and establish the role of CSF NfL as a potential biomarker of preclinical neuropathology, dementia onset and progression, this study was conducted using baseline CSF samples of participants from the Australian Imaging, Biomarkers and Lifestyle study of ageing (AIBL), a prospective longitudinal study initiated in 2006. In this study, we assessed the utility of CSF NfL to: (1) screen individuals with preclinical AD (CH individuals with high brain Aβ, high CSF P-tau, and either high or low CSF T-tau [A+T+/N±]); (2) predict the risk of dementia onset, i.e., risk of developing questionable or very mild dementia (conversation from Clinical Dementia Rating [CDR] 0 to CDR ≥ 0.5 in CH individuals); and (3) to identify individuals who are likely to progress to a state of further cognitive impairment, i.e., predict cognitive decline among individuals with MCI via annual rate of change in cognitive scores (Mini-Mental Status Examination [MMSE] and Clinical Dementia Rating–Sum of boxes [CDR-SB]).

## 2. Methods

### 2.1. Participants

**Figure 1:**
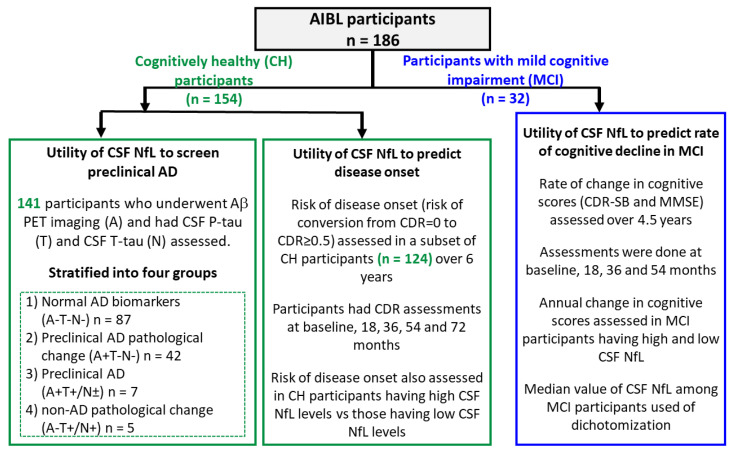
Outline of study protocol and design ATN refers to biomarkers of amyloid pathology (A), tau pathology (T) and neurodegeneration (N). Abbreviations: Aβ: amyloid-β; CDR: Clinical Dementia Rating Scale; CDR-SB: Clinical Dementia Rating–Sum of Boxes; CSF: cerebrospinal fluid; MMSE: Mini Mental State Examination; NfL: neurofilament light; PET: positron emission tomography.

For this study, AIBL participants who agreed to CSF collection were included (*n* = 186, aged ≥ 60 years). Of these, 154 were CH, and 32 had MCI. Participants’ CSF was collected between 2009 and 2016. Participants were clinically classified as MCI or healthy controls/cognitively healthy (CH), using all the available information [20]; MCI was determined based on the criteria of Petersen et al. [21]. Participants were classified as cognitively healthy based on their performance in neuropsychological and cognitive tests. Exclusion criteria include heavy alcohol consumption (exceeding two standard drinks per day for women and four per day for men), previous serious head injury, history of non-AD dementia, current clinical depression, schizophrenia, bipolar disorder, epilepsy, amnesia, Parkinson’s disease, cancer (other than basal cell skin carcinoma) within the last two years, history of stroke, uncontrolled diabetes and withdrawal of consent [20]. Age, sex and apolipoprotein E (*APOE*) allele data were assessed as part of the cohort demographic characterization. The *APOE* genotype was assessed using TaqMan genotyping assays (Life Technologies, Mulgrave, Australia) for rs7412 (Assay ID: C_904973_10) and rs429358 (Assay ID: C_3084793_20). Figure 1 outlines the study protocol and design.

### 2.2. Ethics Approval

The AIBL has been approved by the institutional ethics committees of Austin Health, St. Vincent’s Health, Hollywood Private Hospital and Edith Cowan University. The ethics approval for this project was additionally provided by Edith Cowan University (#17380). All participants provided their written informed consent before participating in the study.

### 2.3. Brain Amyloid-β (Aβ) Imaging and Stratification of Cognitively Healthy Participants

A subset of the CH participants (*n* = 141) underwent brain PET Aβ imaging with different tracers ^11^C-Pittsburgh compound B (^11^C-PiB), and ^18^F labelled compounds ^18^F-florbetapir and ^18^F-flutemetamol. The detailed procedures involving individual ligands/tracers are described elsewhere [22,23,24]. Each tracer has different pharmacokinetic, binding affinities and specific reference regions that are reflected in their distinct standardised uptake value ratio (SUVR) cut-off to distinguish high Aβ (A+) from low Aβ (A−). Therefore, to place the standardised uptake value ratio (SUVR) of the F-18 amyloid tracers on a single continuous scale, the SUVRs were linearly transformed into PiB-like SUVR units called the Before the Centiloid Kernel Transformation (BeCKeT) [25]. Brain Aβ positivity was then determined based on an SUVR/BeCKeT cut-off value of 1.4 [26].

Using the biomarker-guided ATN classification recommended by the National Institute on Aging and Alzheimer’s Association (NIA-AA) research framework (2018) [4], CH participants (*n* = 141) were stratified into the following four groups: (1) normal AD biomarkers (A-T-N-), (2) preclinical AD pathological change (A+T-N-), (3) preclinical AD (A+T+/N±) and (4) non-AD pathological change (A-T+/N+). Cut-off values for T-tau and P-tau positivity (in pg/mL) were determined against AIBL PET-amyloid data using PIB SUVR of 1.4, using a rank order approach described by Li QX et al., 2015 [27] (T-tau 327.55 [60–70 years)], 497.80 (>70 years); P-Tau 65.56 (60–70 years), 82.85 pg/mL [>70 years]).

### 2.4. Cognitive Assessments

A neuropsychological battery was used for the cognitive assessments of the participants [20]. CDR scores were used to assess the utility of CSF NfL to predict dementia onset, i.e., the onset of questionable or very mild dementia (risk of conversion from CDR = 0 to CDR ≥ 0.5) among CH participants. CDR scores were assessed over 6 years, following baseline CSF collection (18, 36, 54 and 72 months). A subset of CH participants (*n* = 124) who had at least three CDR assessments were included for this analysis. Due to potential underestimation/overestimation, CH participants who had less than three CDR assessments were not included. Among these 124 participants, 25 converted from CDR = 0 to CDR ≥ 0.5. MMSE and CDR-SB scores that were assessed at baseline and at follow-up time points (18, 36 and 54 months), were used for disease prognostic analysis among participants with MCI (*n* = 32). MCI participants had an average of three assessments for MMSE and CDR-SB, and each participant had at least two cognitive assessments.

### 2.5. CSF Samples

CSF samples were obtained at two centres (Perth and Melbourne), by the usual procedure of lumbar puncture (LP) according to the protocol outlined by the Alzheimer’s Biomarkers Standardization Initiative [28]. Samples were centrifuged within 2 h, at 2000× *g* for 10 min and then aliquoted into polypropylene tubes (0.5 mL) and stored at −80 °C. Samples underwent one freeze–thaw cycle to aliquot the samples further.

### 2.6. Biomarker Analyses

CSF NfL levels were analysed in duplicate using a commercial ELISA (NF-light; UmanDiagnostics, Umeå, Sweden) following the manufacturer’s protocol. Core CSF biomarkers (amyloid-β 42, Aβ42; total tau, T-tau and phosphorylated tau, P-tau) were analysed in duplicate using enzyme-linked immune sorbent assays (ELISAs): INNOTEST β-AMYLOID _(1–42)_ (Aβ42), INNOTEST hTAU Ag (T-tau), and INNOTEST PHOSPHO-TAU _(181P)_ (P-tau181P) (Fujirebio, Ghent, Belgium).

### 2.7. Statistical Analyses

Statistical analyses were performed using IBM SPSS version 27 (for Microsoft Windows). Cross-sectional differences for continuous variables were assessed with an analysis of covariance (ANCOVA), controlled for age, sex and *APOE* genotype (presence of ε4 allele). Bonferroni corrections were applied for multiple comparisons. Chi-squared tests were used to compare categorical variables. Where required, assumptions of normality were met by transforming the variables using a natural logarithm. The ability of CSF NfL to distinguish CH individuals with preclinical AD (A+T+/N±) from those with normal AD biomarkers (A-T-N-), and preclinical pathological change (A+T-N-), was assessed via a receiver operating characteristic (ROC) curve analysis. The Cox proportional hazards model was used to assess the utility of baseline CSF NfL levels for predicting dementia onset, i.e., the risk of developing questionable or very mild dementia (risk of conversion from CDR = 0 to CDR ≥ 0.5) among CH participants (*n* = 124) over 6 years, controlled for age, sex and *APOE* genotype (presence of ε4 allele). The effect of baseline CSF levels–high vs. low, on risk of dementia onset was assessed by dichotomizing CH participants using an optimum cut-off that provided the maximum value of the Youden index (sensitivity + sensitivity − 1) [29], following the ROC curve analysis to distinguish converters from non-converters. Kaplan–Meier survival curves were generated to illustrate the risk of dementia onset, i.e., the development of questionable dementia over 6 years, in the high CSF NfL group vs. low CSF NfL group. A log rank test was used to compare survival distributions of the groups.

Mixed linear models were used to assess the utility of baseline levels of CSF NfL, as well as the utility of core CSF biomarkers (Aβ42, T-tau and P-tau) for predicting the rate of cognitive decline among participants with MCI over 4.5 years, assessed through the annual change in MMSE and CDR-SB scores. The rate of cognitive decline in MCI participants was assessed as a function of CSF biomarkers by using CSF biomarkers (CSF NfL, Aβ42, T-tau and P-tau) as categorical variables–to assess the effect of high and low baseline CSF measure on rate of cognitive decline. MCI participants were categorized into two groups–groups 1 (low CSF biomarker) and group 2 (high CSF biomarker) based on the 50th percentile value (median) of CSF biomarkers amongst the MCI participants. MMSE and CDR-SB scores were used as dependent variables, and time interval between follow-up assessments as a continuous variable. For all analyses *p* > 0.05 was considered significant and all analyses were controlled for covariates age, sex and *APOE* genotype, given the possible effect of these covariates on outcomes assessed.

## 3. Results

### 3.1. Utility of CSF NfL to Screen Preclinical AD and Predict Dementia Onset

#### 3.1.1. Screening of Preclinical AD

This analysis was carried out in CH participants (*n* = 141) who underwent Aβ PET imaging (A) and had their CSF P-tau (T) and T-tau (N) assessed (Figure 1). Differences in mean levels of CSF NfL were assessed among the following four groups of CH participants: (1) normal AD biomarkers (A-T-N-, *n* = 87), (2) preclinical AD pathological change (A+T-N-, *n* = 42), (3) preclinical AD (A+T+/N±, *n* = 7) and (4) non-AD pathological change (A-T+/N+, *n* = 5). The results are shown in Table 1. CSF NfL levels were found to be significantly elevated in the preclinical AD group compared to the normal AD biomarkers group (*p* < 0.001; area under the ROC curve [AUC] = 0.79, CI 0.60–0.98, *p* = 0.011). CSF NfL levels were also significantly elevated in the preclinical AD group compared to the preclinical AD pathological change group (*p* = 0.002, AUC = 0.75, CI 0.53–0.96, *p* = 0.040). CSF NfL levels were higher in the preclinical AD group compared to non-AD pathological change, though not significantly.

#### 3.1.2. Prediction of Dementia Onset

**Figure 2:**
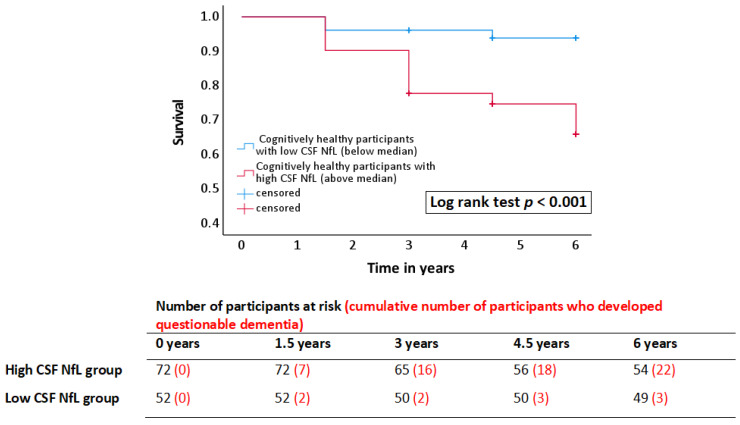
Kaplan–Meier curves illustrating risk of dementia onset (onset of questionable or very mild dementia; conversion to CDR ≥ 0.5) among cognitively healthy participants as a function of CSF NfL over 6 years. Cognitively healthy (CH) participants (*n* = 124) were categorized into two groups–high and low CSF NfL—using a cut-off value of 674 pg/mL, that gave maximum value of Youden index (sensitivity + sensitivity − 1), following the ROC curve analysis to distinguish converters from non-converters. Figure 2 illustrates the probability of developing questionable over 6 years in CH participants having high vs. low CSF NfL. Participants with high CSF NfL had a higher probability of developing questionable dementia (log rank test *p* < 0.001). The total number of participants who developed questionable dementia over 6 years were significantly higher in the high CSF NfL group compared to those in the low CSF group (*p* < 0.001). Abbreviations: CSF: cerebrospinal fluid; CDR: Clinical Dementia Rating; CSF: cerebrospinal fluid; NfL: neurofilament light; ROC: Receiver Operating Characteristic.

Cerebrospinal fluid NfL levels were significantly elevated in participants who converted from CDR = 0 to CDR ≥ 0.5, as compared to non-converters (*p* = 0.045; Table 2), after controlling for age, sex and *APOE* genotype. A Cox proportional hazards model was used to assess the utility of CSF NfL for predicting dementia onset among CH participants (risk of conversion from CDR = 0 to CDR ≥ 0.5). CSF NfL levels (Z scores) significantly predicted the risk of conversion from CDR = 0 to CDR ≥ 0.5 over 6 years, with a hazard ratio (HR) of 1.60 (CI 1.03–2.48, *p* = 0.038), after controlling for age, sex and *APOE* genotype. Participants (CH) whose CSF NfL levels were above the optimum cut-off (>674 pg/mL, determined via Youden Index; sensitivity 0.88 and specificity 0.50) were at a higher risk of dementia onset (HR 4.77, CI 1.31–17.29, *p* = 0.018). The Kaplan–Meier survival curves shown in Figure 2 illustrate that higher CSF NfL levels are predictive of a higher risk of dementia onset (onset of questionable or very mild dementia) among CH participants. Participants with high CSF NfL had a lower cumulative survival probability or a higher cumulative hazard of developing questionable dementia over 6 years, compared to those with low CSF NfL (*p* < 0.001). The high CSF NfL group had 22 CH participants who converted to CDR ≥ 0.5 or developed questionable dementia over 6 years, while the low CSF NfL group had three such participants (*p* < 0.001; Figure 2).

### 3.2. Utility of CSF NfL for Predicting Rate of Cognitive Decline in MCI

Cerebrospinal fluid NfL levels (mean levels, pg/mL) were significantly elevated in participants with MCI vs. CH (*p* < 0.001) after controlling for covariates age, sex and *APOE* genotype (MCI 1017 pg/mL [SD 450.93]; CH 751 pg/mL [253.29]). The prognostic utility of NfL was assessed using mixed linear models, for predicting the rate of cognitive decline in participants with MCI over 4.5 years, assessed through the annual change in MMSE and CDR-SB scores. For comparison, a disease prognostic analysis was also carried out for core CSF biomarkers (CSF Aβ, T-tau and P-tau). MCI participants (*n* = 32) were dichotomized into groups (low and high CSF measure) based on the 50th percentile of CSF measures (NfL 807 pg/mL, Aβ42 650 pg/mL, T-tau 274 pg/mL, P-tau 58 pg/mL). The annual change in MMSE and CDR-SB scores was assessed in the two groups after controlling for age, sex and *APOE* genotype (Table 3).

**Figure 3:**
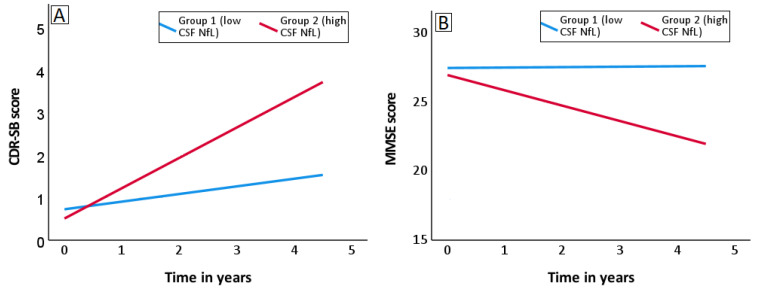
Prediction of cognitive decline in participants with MCI as a function of CSF NfL over 4.5 years. Participants with MCI (*n* = 32) were categorized into two groups–high and low CSF NfL—using a cut-off value of 807 pg/mL (50th percentile). Trend lines depicted in the above graphs are regression lines. (**A**) Rate of change in CDR-SB scores in high and low CSF NfL groups. (**B**) Rate of change in MMSE scores in high and low CSF NfL groups. The rate of decline in cognition (rate of increase in CDR-SB and rate of decrease in MMSE) was higher in the group with higher baseline CSF NfL. Abbreviations: CSF: cerebrospinal fluid; CDR-SB: Clinical Dementia Rating Scale–Sum of Boxes; CSF: cerebrospinal fluid; NfL: neurofilament light; MCI: mild cognitive impairment; MMSE: Mini Mental State Examination.

For CSF NfL, the annual change in CDR-SB (rate of increase) and annual change in MMSE (rate of decrease) was higher in group 2 (high CSF NfL group). A higher rate of decrease in MMSE and a higher rate of increase in CDR-SB indicates a higher rate of cognitive decline. The annual change in CDR-SB was 0.68 points in the high CSF NfL group (*p* < 0.001), and 0.19 in the low CSF NfL group (*p* = 0.003). The annual change in MMSE was −0.99 points in the high CSF NfL group (*p* = 0.001), and −0.12 in the low CSF NfL group (*p* = 0.372). Figure 3A and 3B depict the rate of change in CDR-SB and MMSE scores in high and low CSF NfL groups. Similar trends were noted for core CSF biomarkers, further confirming the potential of CSF NfL to predict rate of cognitive decline in MCI. For CSF T-tau, the annual change in CDR-SB was 0.70 points in the high CSF T-tau group (*p* < 0.001), and 0.20 in the low CSF T-tau group (*p* = 0.002). The annual change in MMSE was −1.03 points in the high CSF T-tau group (*p* = 0.003), and −0.14 in the low CSF T-tau group (*p* = 0.214). For CSF P-tau, the annual change in CDR-SB was 0.78 points in the high CSF P-tau group (*p* < 0.001), and 0.18 in the low CSF P-tau group (*p* = 0.003). The annual change in MMSE was −1.31 points in the high CSF P-tau group (*p* < 0.001), and −0.04 in the low CSF P-tau group (*p* = 0.734). For CSF Aβ42, as expected, the annual change in cognitive scores was higher in the low CSF Aβ42 group. Lower levels of CSF NfL, T-tau, P-tau and higher levels of CSF Aβ42 did not indicate the rate of change in MMSE scores. Therefore, further studies are required to establish the association.

## 4. Discussion

Cerebrospinal fluid NfL is a biomarker of neurodegeneration whose levels are elevated in AD and associate with central neuropathological changes–tauopathy, amyloidopathy and brain atrophy [6,7,8,9,10,16]. Evidence of Aβ (A), and P-tau (T) or T-tau (N) positivity in CH individuals is indicative of preclinical AD [4]. We noted that CSF NfL levels were significantly elevated in the preclinical AD group (A+T-/N±) and distinguished preclinical AD participants from those with normal AD biomarkers (A-T-/N-) with an acceptable diagnostic accuracy (AUC 0.79). A recent study by Andersson et al., (2020) found that CSF NfL levels were significantly elevated in CH participants classified as Aβ+ (A+) based on Aβ42/Aβ40 ratio cut-off [10]. Participants were also classified as neurodegeneration negative or positive (N− or N+) based on cortical thickness of AD-susceptible temporal regions [10]. CSF NfL levels were elevated in CH participants classified as A+N+ and A+N− (*p* = 0.00125) compared to A-N- for both groups) and much higher in A+N+ (1.66 times higher) [10]. Findings from this study and our study support that CSF NfL can be used as a potential screening biomarker for preclinical AD. Given that we observed a fair diagnostic accuracy of CSF NfL for identifying cases preclinical AD, NfL can form a highly accurate diagnostic classifier in conjunction with biomarkers associated other pathological changes.

We found significantly elevated baseline levels of CSF NfL in CH individuals who developed questionable dementia or very mild dementia over 6 years. In addition, the elevated levels predicted the risk of developing questionable dementia. We noted that individuals with CSF NfL levels above optimal threshold are at much higher risk of dementia onset. Future studies with a larger sample size should test our findings by classifying CH participants using a specific threshold, or into tertiles or quartiles. Since AD is a neurodegenerative disease, the preclinical stage could be associated with an initial stage of neurodegeneration whereby clinical symptoms are not evident, but neurodegeneration exits, though below a certain threshold. Upon disease development or onset, the evolution of the neurodegenerative process in the brain could be equated to the rate of change in the severity of clinical symptoms or vice versa [30]. As a biomarker of neurodegeneration, CSF NfL levels could reflect such neurodegenerative changes, as well as the associated change in clinical symptoms. Our results indicate that CSF NfL levels predict the rate of cognitive decline among individuals with MCI. The rate of cognitive decline as measured by the rate of increase in CDR-SB and rate of decrease in MMSE was higher in MCI participants with CSF NfL above the threshold (median level). Similar trends noted for core CSF biomarkers confirm the potential of CSF NfL to predict cognitive decline in individuals with MCI. Further studies are needed to validate the potential of CSF NfL to predict cognitive decline in individuals with MCI. Previous studies confirm that higher levels of CSF NfL predict a higher rate of cognitive decline, as well as the conversion of MCI to AD [6,31]. Therefore, our results indicate that brains of individuals with MCI, with high baseline levels of CSF NfL, undergo faster evolution of neurodegenerative processes, resulting in faster cognitive decline.

Our results indicate that CSF NfL could be used as a pathophysiological biomarker of stage-associated neurodegeneration. The identification of stage-associated changes will help in targeting a focused therapy, which can be applied at an early stage, defined by specific pathological changes. An early and targeted intervention can help in reducing the risk of developing dementia or can postpone development. In addition, assessments of CSF NfL levels can help in enriching the clinical trials, by including individuals who are likely within the early phase of the AD continuum, based on their biomarker profile [32]. Moreover, outcomes of clinical trials can be ascertained with much more reliability, using CSF NfL levels as an inclusion criterion, as well as an end point measure, i.e., a treatment response biomarker to evaluate the effectiveness of therapeutic interventions targeted at aberrant biochemical pathways promoting axonopathy. Olsson et al. (2019) demonstrated the utility of CSF NfL as a treatment response biomarker in spinal muscular atrophy [33].

A small cohort of MCI participants represents the major limitation for our study. Given the small number of participants with MCI, we separated them using the 50th percentile or median value of CSF NfL, for disease prognostic analysis. A relatively small number of CH participants could present another limitation. Therefore, the estimated risk of dementia onset among CH participants (high CSF NfL group vs. low CSF NfL group), and rates of cognitive decline among participants with MCI could have been overestimated. Findings should be tested in larger cohorts by stratifying participants into terciles or quartiles, or dichotomizing them using an optimal cut-off. The utility of CSF NfL to predict the risk of dementia onset should also be assessed in longitudinal studies using CH participants who convert to MCI, and to predict disease progression using MCI participants who progress to AD.

In conclusion, CSF NfL can be used as a pathophysiological biomarker which reflects early neurodegeneration, and as a prognostic biomarker which reflects downstream neurodegenerative changes along the AD continuum. As a diagnostic biomarker, it can help to screen preclinical AD and predict the risk of dementia onset. Thereby, it can help to optimize the drug development process by targeting the right drug candidate, at the right biochemical pathway and at the right stage. As a prognostic biomarker, it can be used as a treatment response biomarker for interventions under trial that aim at slowing down disease progression by combating axonopathy. A biomarker-based diagnostic algorithm, which includes CSF NfL, could be developed to screen preclinical AD with high accuracy. To increase the clinical applicability of NfL, future studies should explore the potential of plasma NfL for screening preclinical AD, predicting the risk of dementia onset among CH participants, and the rate of cognitive decline in participants with MCI.

## Figures and Tables

**Table 1:** Demographic and biomarker characteristics of cognitively healthy participants segregated using the biomarker guided ATN classification framework.

Participant Characteristics	Cognitively Healthy (*n* = 141)			
	Normal AD Biomarkers (A-T-N-)	Preclinical AD Pathological Change (A+T-N-)	Preclinical AD (A+T+/N±)	Non-AD Pathological Change (A-T+/N+)
**Demographics**				
Number of participants	87	42	7	5
Sex M/F (% females)	35/52 (60%)	24/18 (43%)	2/5 (71%)	3/2 (40%)
Age at LP in years	72 (5.48)	74 (4.81)	70 (4.63)	76 (9.07)
*APOE* ε4 allele not present/present (% of ε4 carriers)	74/13 (15%)	23/19 (45%) ^$^	5/2 (29%)	4/1 (20%)
**Neurofilament light**				
CSF NfL pg/mL	709 (241.27)	775 (223.58)	1070 (394.11) ^%,§^	885 (355.46)

ATN refers to biomarkers of amyloid pathology (A), tau pathology (T) and neurodegeneration (N). This analysis was performed on a subset of cognitively healthy participants (*n* = 141) who underwent Aβ PET (A) imaging and had measurement of CSF levels of P-tau (T) and T-tau (N). The values of NfL in the table represent raw means (SD) unless indicated. CSF NfL values were transformed using the natural logarithm and differences were compared among the groups using ANCOVA after controlling for age, sex and *APOE* genotype status. Demographic differences were assessed using Chi-squared tests or ANOVA. ^$^ *p =* 0.001 vs. Normal AD biomarkers group, ^%^ *p <* 0.001 vs. Normal AD biomarkers group, ^§^ *p* = 0.002 vs. Preclinical AD pathological change. Abbreviations: Aβ: amyloid-β; *APOE*: apolipoprotein E; CSF: cerebrospinal fluid; LP: lumber puncture; NfL: neurofilament light; PET: positron emission tomography; P-tau: phosphorylated tau; T-tau: total tau.

**Table 2:** Demographic characteristics and CSF NfL levels among cognitively healthy participants who converted to CDR ≥ 0.5 from CDR = 0 and non-converters.

Participant Characteristics	Participant Groups		*p*-Value
	Converters	Non-Converters	
**Demographics**			
Number of participants (*n* = 124)	25	99	
Sex M/F (% females)	14/11 (44%)	42/57 (58%)	0.223
Age at LP in years	75 (4.82)	72 (5.68)	0.011
*APOE* ε4 allele not present/present (% of ε4 carriers)	18/7 (28%)	77/22 (22%)	0.542
**Neurofilament light**			
CSF NfL pg/mL	877 (256.24)	714 (252.29)	0.045

This analysis was performed on a subset of cognitively healthy (CH) participants (*n* = 124) who had a minimum of three CDR assessments over 6 years (baseline, 18, 36, 54 and 72 months). The values for CSF NfL represent raw means (SD) unless indicated. CSF NfL values were transformed using the natural logarithm and differences were compared among the groups using ANCOVA after controlling for age, sex and *APOE* ε4 genotype. Demographic differences were assessed using Chi-squared tests or independent samples t-test. Abbreviations: AD: Alzheimer’s disease; *APOE*: apolipoprotein E; CDR: clinical dementia rating; CSF: cerebrospinal fluid; LP: lumber puncture; NfL: neurofilament light.

**Table 3:** Annual rate of change in MMSE and CDR-SB scores in participant groups with MCI–classified based on high and low measure of CSF biomarkers.

CDR-SB				
Biomarker	Group 1 (low CSF Biomarker)	*p*-Value	Group 2 (High CSF Biomarker)	*p*-Value
CSF NfL	0.19	0.003	0.68	<0.001
CSF Aβ42 *	0.65	<0.001	0.24	0.004
CSF T-tau	0.20	0.002	0.70	<0.001
CSF P-tau	0.18	0.003	0.78	<0.001
**MMSE**				
**Biomarker**	**Group 1** **(low CSF Biomarker)**	***p*-Value**	**Group 2** **(High CSF Biomarker)**	***p*-Value**
CSF NfL	−0.12	0.372	−0.99	0.001
CSF Aβ42 *	−1.17	<0.001	−0.02	0.875
CSF T-tau	−0.14	0.214	−1.03	0.003
CSF P-tau	−0.04	0.734	−1.31	<0.001

Participants with MCI were dichotomized based on the 50th percentile value for each CSF biomarker. Participants had four cognitive assessments over 4.5 years, with an average of three assessments (baseline, 18, 36 and 54 months). Results are depicted graphically in Figure 3. * For CSF Aβ42, as expected, the annual change in cognitive scores was higher in the low CSF Aβ42 group. Abbreviations: AD: Alzheimer’s disease; Aβ: amyloid-b; CDR-SB: Clinical Dementia Rating Scale–Sum of Boxes; CSF: cerebrospinal fluid; NfL: neurofilament light; MCI: mild cognitive impairment; MMSE: Mini Mental State Examination; P-tau: phosphorylated tau; T-tau: total tau.

## Data Availability

Data may be provided on request by the corresponding author.
